# A collaborative working environment for small group meetings in Second Life

**DOI:** 10.1186/2193-1801-2-281

**Published:** 2013-06-27

**Authors:** Cintia RC da Silva, Ana Cristina B Garcia

**Affiliations:** Departamento de Computação, Polo de Rio das Ostras, Universidade Federal Fuminense, Rio das Ostras, RJ, Brazil; Departamento de Ciência da Computação, Instituto de Computação, Universidade Federal Fuminense, Niteroi, RJ, Brazil

**Keywords:** CSCW, Second life, SLMeetingRoom, Work meetings, Groupware, Virtual work places

## Abstract

This paper presents the *SLMeetingRoom*, a virtual reality online environment to support group meetings of geographically dispersed participants. A prototype was developed to demonstrate the feasibility of the approach using the Second Life platform. Ten additional components had to be added to Second Life environment to support group work essential activities such as participants’ **communication**, tasks’ and participants’ **coordination**, participants’ **collaboration** and work evolution’s **perception**. Empirical studies, both pilot and experiment, were developed comparing four different meeting settings: face-to-face, videoconference, stand Second Life and *SLMeetingRoom*. The study involved graduate students enrolled in the Interface and Multimedia discipline at the Fluminense Federal University (UFF) in Brazil. Results indicated that groups working within *SLMeetingRoom* environment presented similar results as face-to-face meeting as far as sense of presence is concerned and with low cognitive effort. Task completion and degree of participation were not affected by the meeting set up. It was concluded that Second Life, in conjunction with the *SLMeetingRoom* components, is a good tool for holding synchronous remote meetings and coexists with other electronic meeting technologies.

## Introduction

When people act in groups the resulting synergy usually produces better results than work undertaken individually because together they can seek ideas and information to help resolve problems. When performing a task, a group may split the activities and attribute responsibilities to them, however there is always the need, from time to time, to get together in meetings (Olson et al., 1993).

Meetings are considered the only effective mechanism for problem solving and consensus building (De Lucia et al., 2008). Meetings are a crucial part of collaborative work and their aim is to enable a group to achieve its objectives in an efficient fashion (Nunamaker et al., 1991).

The emergence of web 3.0 and 3D virtual environment technologies has opened up new possibilities for holding virtual meetings (De Lucia et al., 2008; Olivier and Pinkwart, 2007; Harry and Donath, 2008). 3D virtual environments, such as Second Life (SL), are interesting media (simulated and shared) for holding meetings with geographically distributed participants. SL has emerged as an environment for interactions between geographically distributed people, arousing the interest of organizations like IBM, Intel and NASA that use it to hold virtual meetings between distributed employees, subsidiaries or customers saving space allocation and travel time (De Lucia et al., 2008; SL, 2010). The academic community uses SL as an educational tool to facilitate student and teacher immersion (Santoro et al., 1999; Rovere and Zago, 2007), while the research community believes in the possibility of developing professional activities within 3D virtual environments (Bessiere et al., 2009), as well as studying SL as a social network (Harris et al., 2000), and using it as a research platform in the social science area (Varvello and Voelker, 2010).

The problem addressed here is that, despite the large number of platforms currently on offer, such as chats, videoconferencing and electronic meeting system (EMS), there is no suitable solution for remote meetings in terms of communication, coordination, cooperation and perception.

3D virtual environments like SL are a good proposal, but do not as yet possess the necessary functionalities for supporting the vital activities of remote meetings. According to (Ellis et al., 1991; Grudin, 1994) the vital activities in a remote meeting that need support are: scheduling of commitments and events, task monitoring, decision-making, voting, information storage and perception. In order to resolve the problem put forward by this research we propose the creation of a model of environment to support the basic activities performed during work meetings. This is followed by the postulation of two mutually exclusive hypotheses:

*H*0(*null hypothesis*): A group using a meeting environment implemented according to the *SLMeetingRoom* model is no different from a group using only Second Life and traditional videoconferencing systems.

*H*1(*alternative hypothesis*): A group using a meeting environment implemented according to *SLMeetingRoom* model should, at the end of the meeting, show a greater degree of task completion, participation, sense of presence and lower cognitive effort than groups using only Second Life and traditional videoconferencing systems.

The study was conducted using the comparative research method whose aim is to compare experimental and control groups through the random allocation of participants (Marczyk et al., 2005). The comparative experimental study was undertaken in two stages: pilot study and experiment. It involved a set of task-oriented work meetings with groups working together using different environments. Pilot study and experiment participants were composed of students of the Federal Fluminense Universitys (UFF) Interface and Multimedia discipline, whose basic profile can be summed up as follows: young people aged between 21 and 31 of both sexes and Brazilian nationality, with medium/advanced experience in informatics and the internet, all studying for a masters degree. The participants did not receive any remuneration, but the course grades acted as an incentive for participation. The analysis of the objective measures was performed using statistical techniques such as summary measures (mean, variance, standard deviation and percentage), regression and correlation. Hypothesis testing was used as a mechanism to provide statistical evidence of differences between experimental groups. In order to compare the groups the research used the Jonckheere-Terpstra test JT (Hollander and Wolfe, 1973), a non-parametric test that verifies differences between ordered treatments. In this test, which is used in the case of problems with more than two samples, the alternative hypothesis can be expressed as: τ1 ≤ τ2 ≤ … ≤ τ*n*, or τ1 ≥ τ2 ≥ … ≥ τ*n*, with at least one of the inequalities, where ‘i’ denotes the effect of the i-nth treatment (experimental group). A significance level of 5% was adopted as a decision criterion. In order to guarantee the experiments internal validity, we controlled the experiments duration, participants profile, participants selection, task scope and observation settings to avoid bias effects caused by testing, instrumentation, regression, selection, mortality and interaction effects, which include actions such as the random allocation of participants, their age group, educational level and informatics/internet experience. We were not able to control factors related to history and testing. Proximity to exams and final studies of other disciplines may have affected participants motivation and responses to the questionnaire applied at the end of each meeting. Results are very promising indicating that *SLMeetingRoom* brings a potential leverage as an environment for group work whenever distance is an issue. Although the evidences provided by our experiments, it is necessary to develop a more extensive experiment with more groups to prove our hypothesis.

## Theoretical references

For quite a long time geographic distance constituted the major selection criteria for the formation of the groups. On the other hand, the globalization of work drove the development of technology to support group interaction using computers, smoothing the challenge posed by distance. Computer Supported Cooperative Work (CSCW) is a research area focused on issues related to understanding how technology can provide support to enable individuals to work together to accomplish a common goal (Ellis et al., 1991).

Face-to-face meetings are the ideal form of group interaction for minimizing communication misunderstandings and maximizing individual sensemaking. They are regarded as the gold standard for group interaction (Bessiere et al., 2009; Beaudouin-Lafon, 1999).

The common workspace brings clear benefits to work meetings, such as improved participant awareness of the issues and decisions that matter to all, participants’ motivation to accomplish their goals, task follow-up, participant accountability for failures, task coordination and productivity (Olson et al., 1998). Communication though gestures, eyesight, posture as well as having access to shared artifacts (screens, models, panels, etc.) reinforce the advantages of on-site meetings (Beaudouin-Lafon, 1999). However, there are some drawbacks, such as the high costs associated with getting people together, the excessive attention to minor issues, individual anxiety regarding how ideas will be received (Nunamaker et al., 1991), individual tension associated with being a speaker (difficulties regarding eloquence and the exposition of ideas)(Dickey-Kurdziolek et al., 2010) and lack of good meeting records. These problems often result in less productive meetings (Nunamaker et al., 1991).

Many authors have signaled out communication mediated by video, also known as videoconferencing, as the natural substitute for face-to-face communication (Bly et al., 1993; Nguyen and Canny, 2007; Yamashita et al., 2008). Videoconferencing systems constitute excellent environment for CSCW as they facilitate communication, increase levels of participation (Bietz, 2008) and reduce travel-associated costs (Beaudouin-Lafon, 1999). However, it is considered an invasive solution that, in a certain sense, restricts privacy during interactions (Cherubini et al., 2008), requires special meeting room configurations and imposes high maintenance costs (Townsend et al., 1996), with remote participants sitting around a worktable (Yamashita et al., 2008).

The limitations of videoconferencing systems for holding distributed meetings have boosted the emergence of electronic meeting systems (EMS) (Noll, 1976; Egido, 1988). EMS was developed in an attempt to overcome the lack of systems designed specifically to support collaboration between groups in virtual meetings seeking meeting effectiveness, task efficiency and participant satisfaction (Nunamaker et al., 1991). The advantages of EMS systems lie in their support for communication, through brainstorming, idea organization and voting tools; support for task coordination through shared agendas and schedules tools; support for cooperation, through work area sharing tools; and support for perception, through participant activity feedback tools. Although EMS systems cover all essential requirements for collaboration, studies show that they have not been widely adopted. They have many limitations, such as high Internet bandwidth consumption, maintenance and installation complexity, people resistance to adapting to new meeting behavior and interaction protocol, and difficulty in measuring benefits (Pervan et al., 2004).

3D virtual environments appeared after the advent of EMS and they are gradually becoming more commonplace as everyday tools for communication, socialization and in meetings (De Lucia et al., 2008; Olivier and Pinkwart, 2007; Harry and Donath, 2008). In 3D virtual environments used for social interaction and communication we found, amongst others: There (Brown and Bell, 2004), Active World (World, 2009) and Second Life (SL 2010). The most popular of interactive metaverse is SL, a virtual reality environment shared among remote web users.

According to (Olivier and Pinkwart, 2007), SL covers most of the essential aspects of synchronous communication that is so important in the CSCW area, such as direct chat, instantaneous messages, voice conversations, gestures, internal groups, etc. It also makes it possible to expand the limits of remote interaction through 3D models that offer excellent potential for new forms of interaction, cooperation and socialization. Participant immersion in the interaction context constitutes the strong point of SL that generates a sense of co-location, simulating a feeling of being physically co-located, making an environment possible and favorable for holding work meetings, given that it has the characteristics of face-to-face and remote meetings.

Second Life has shown excellent potential for the development of collaborative activities because its real world characteristics allow the user to be immersed in activities and feel part of the environment. Like EMS, SL fulfills the requirements of collaboration. Communication activities are efficiently developed and perception and cooperation are supported by the quality of 3D interactions and, on a smaller scale, ensure coordination through internal groups.

## The *SLMeetingRoom* model

**Figure 1:**
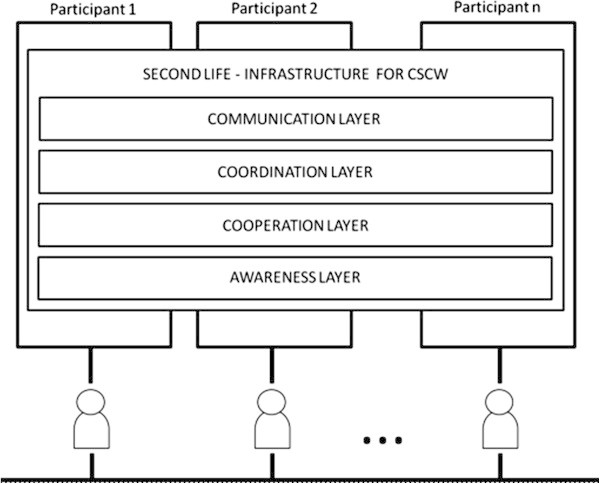
**SLMeetingRoom Model.**

Collaboration can be seen as a combination of communication, coordination, and cooperation activities allied to perception. This set of three activities is known as the 3C collaboration model. This model determines the essential requirements for effective collaboration between members of a group and is seen as the objective of groupware (Ellis et al., 1991), as illustrated in Figure 1.

The *SLMeetingRoom* model is a meeting environment model for work groups in which participants are geographically distributed. According to the *SLMeetingRoom* model, the layers represent required services that the environment must comply. The SL basic layer implicitly provides the distributed communication layer. The other layers can be understood as plug-ins to the SL layer. The users who enter the meeting room supported by the *SLMeetingRoom* model will have access to all layers. The components act as service providers in the layers and can operate in several layers at the same time.

There is a dependency relationship among the layers in the *SLMeetingRoom* model. Communication generates commitments for coordination. Efficient coordination facilitates cooperation activities and perception supports communication and coordination. Each layer is usually analyzed separately but they operate in an integrated fashion during group work.

The choice of the necessary components for the *SLMeetingRoom* model was based on three pilot studies that used SL to hold meetings. Through these experiments we observed that the greatest difficulty in using SL to hold meetings, lay in the lack of the even the most basic tool to support work meetings in order to conduct activities aimed at achieving the groups objectives.

Table 1 lists all ten the components that enriched SL environment to support group work. The second column illustrates the main functionality and the third column presents secondary functions.

**Table 1:** **Components of the*SLMeetingRoom*model**

Component	Main function	Extra functions
Whiteboard	Communication	Cooperation
Meeting Agenda	Coordination	
Schedule	Coordination	Perception
Repository	Communication	Cooperation
Argument Model	Cooperation	
Voting Box	Cooperation	
Gesture’s panel	Communication	Perception
Presence List	Perception	Coordination
Chronometer	Coordination	Perception
Social Proxy	Perception	Coordination

## Comparative experimental study

### Pilot study

#### Overview

The pilot study was composed of a series of work meetings, with groups working together in four different settings: Face-to-Face, Videoconference, SL without *SLMeetingRoom* and SL with *SLMeetingRoom*. The goal was to assess whether the proposed model supported the hypothesis that guided this research.

The pilot study was carried according to the University’s guidelines, concerning software evaluations, does not require previous consent. Nonetheless, formal written consent were obtained from all participants before the experiment started. It was emphasized that, even though there was no harm in participating in the experiment, they could leave at anytime. Their participation was anonymous and we were looking for the aggregate behavior of the group. The experiment was in compliance with the Helsinki declaration.

#### Participants

Twelve Computer Science master’s students from Fluminense Federal University (UFF), enrolled in the Interface course. Participation was part of the course activities. The students already knew each other from previous courses, but they had never worked together. The participants did not receive any remuneration. The course score acted as an incentive for participation.

#### Task

The task was the design of a web application interface for an optics store to be used by opticians. The group had to develop a user model, task model and a storyboard for the interaction.

#### Duration

The groups were monitored over a period of a fortnight. Each group held four meetings that lasted a maximum of one hour. Only one of the groups exceeded the stipulated time by two minutes. Each group took part in four meetings (a total of sixteen) and used one of the proposed meeting scenarios. The product of each group was assessed by the responsible lecturer and scores were incorporated into students overall course scores.

#### Group configuration and work spaces

Four groups were formed with three participants allotted to each on a random basis (through a draw). As already mentioned, each group held the projects meetings in one of the four work environments, also allotted randomly.

The first group, in the *Face-to-Face* condition met in a meeting room in UFFs ADDlabs (Active Design and Intelligent Design Laboratory), composed of a table, chairs and a network access point for personal laptops. The meetings were video-recorded.

The second group using the *Videoconference* environment (chat, audio and active video), used a videoconferencing client called ooVoo (http://www.oovoo.com) (Wang et al., 2009). The ooVoo is a free videoconferencing system that can be used by up to five people simultaneously. It is composed of a group of functionalities such as textual chat, video chat, work area sharing and file sending, amongst others (ooVoo 2010). The conversations were audio-recorded and chat logs recorded. None of the participants had any previous experience with SL or the experiment.

The third group, within the *SL wihout SLMeetingRoom* environment, met in a meeting room constructed in SL on the ADDLabs Island. The group did not use any component to support the meeting process apart from a table, chairs, chat and audio. Conversations were audio-recorded and the chat logs recorded.

**Figure 2:**
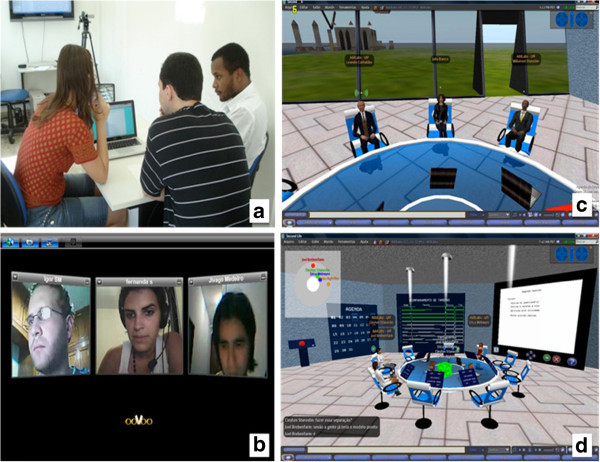
**Work environments and their respective groups working in the meeting rooms.** (**a**) Face-to-Face Group; (**b**) Videoconference Group; (**c**) SL without SLMeetingRoom Group; (**d**) SL with SLMeetingRoom.

The fourth group, within the *SL with SLMeetingRoom* environment, also met in a meeting room constructed in SL on the ADDLabs Island, but used the *SLMeetingRoom* model to support the meeting process. As in the other cases, the meetings were video-recorded and chat logs recorded. Figure 2 shows the participants of the four groups at work in their respective meeting rooms.

#### Process

Each group held four meetings (totaling sixteen) and used one of the environments proposed. Each meeting lasted an hour. One of the project rules stipulated that each group could only use one of the environments as a platform for holding meetings. Participants were not allowed to meet to take decisions related to the project outside their work environments. Before starting pilot study meetings participants were required to answer a Participant Profile Questionnaire based on (Dumas and Redish, 1999). They were asked to fill in and sign a Declaration of Consent (TCLE), adapted from (PROAC-UFF, 2010), whose aim was to obtain the consent of participants to take part in the experiments. Participants were then asked whether they agreed with the terms of the Authorization for the Use of Picture and Declarations, adapted from (Unigranrio 2010), whose aim was to seek authorization for the use of pictures and declarations captured during the experiments. At the end of each meeting participants were asked to respond to a Post-meeting questionnaire, based on a 5-point Likert scale (0-totally disagree and 4-totally agree), in which they were asked about their impressions while using the respective meeting room environments. There was an observer in all meetings. His role was to video record the meetings, to time stamp the scenes and to take notes of unexpected events. The observer, however, did not interact in any way with group participants.

#### Data collection

Pilot study data was collected through observations of the sixteen meetings held which were filmed, audio-recorded and whose chat logs were also registered. Approximately three hours of film was collected during the Face-to-Face meetings. Approximately three hours of audio-recordings were collected during meetings held using the Videoconference environment. In the case of the meetings held using the SL without *SLMeetingRoom* environment, which used a voice communication system, the research collected approximately four hours of audio-recordings. And finally, in the case of meetings held in the SL with *SLMeetingRoom*, environment, in which the group used a text communication system, the research collected approximately 29 sheets of typed text (6000 words). 6 hours/day, 5 days/week during 2 months, or a total of 240 hours, were spent performing a manual analysis of the data.

This research considered four evaluation criteria:

*Task Completion:* this assessment criterion measures the proportion of tasks on the meeting agenda completed during the stipulated meeting duration of one hour. Two measures were used for this task completion assessment criterion: (a) the relation between the number of tasks on the agenda r (NTAr) and the number of tasks performed during a meeting r (NTRr) related to the meetings duration (Tr); and (b) a subjective measure to evaluate participants perception of the meetings effectiveness in addressing the relevant tasks, derived from participants answers in the post-meeting questionnaire based on (Bastea-Forte and Yen, 2007), in which participants were asked whether they had managed to address all the topics on the meetings agenda and if the time allotted for the meeting was sufficient to fulfill the entire agenda.

*Participation:* this criterion evaluates the group’s degree of participation during a meeting. Two measures were used for this criterion: an objective one based on the participants number of conversation turns during a meeting (NTCi) and the duration of participants conversation turns i (DTCi); and a subjective measure based on questions regarding individual and group participation in the post-meeting questionnaire based on (Bastea-Forte and Yen, 2007) and (DiMicco et al., 2004). The conversations were transcribed manually including pauses, silences and other interruptions. Duration was measured in seconds. In the case of groups that used audio communication, conversation turn duration was measured by timing participant speech time. For the group that used text as a communication channel, turn duration was measured by timing how long participants took to type when formulating their speech.

*Cognitive Effort:* this assessment criterion measures the cognitive effort (ease or difficulty) involved in holding meetings in each environment. This criterion was evaluated through a series of subjective questions in the post-meeting questionnaire based on the NASA-TLX MANUAL, a free tool for performing a subjective assessment of a particular users workload (Hart and Staveland, 1988). The application of the NASA-TLX tool is based on six subscales: mental demand (perceptive activity required by a task), physical demand (difficulty to execute a task), temporal demand (pressure due to the time in which a task must be concluded) personal performance (degree of success in performing the task), effort (needed physical and mental work to execute a task at a specific level) and level of frustration (how the continuous stress process is correlated with the conclusion of the task).

*Sense of presence:* the aim of this assessment criterion is to measure participants’ degree of presence perception, using each of the work environment settings. Given that the sense of presence is a subjective experience (Sheridan, 1992), this criterion was measured through answers to questions in the post-meeting questionnaire based on (Kramer et al., 2006), (Usoh et al., 2000) and (Witmer and Singer, 1998). In order to avoid problems that are inherent to subjective measures, such as anchoring effects, imprecise memories and the inability to describe the subjective variations of presence, this research also used an objective measure. The objective criterion was based on (Kramer et al., 2006), who proposed a method for measuring sense of presence based on dialogues linguistic characteristics, i.e., if a person speaks about a remote space in the same way he speaks about the local space, we may infer that he feels present in the remote space. In (Kramer et al., 2006), the authors analyzed conversations in order to identify the local and remote deixis^a^ , pronouns (I, we, you, he/she) and verb tense, which may induce sense of presence in participants. They then related the use of specific linguistic characteristics to the senses of presence reported in the questionnaire.

### Experiment

The hypothesis test results provided evidence in favor of the *SLMeetingRoom* model in the case of the cognitive effort and sense of presence criteria. These results encouraged us to better understand the gains to be achieved by using a virtual environment prepared to support meetings, i.e. the benefits of implementing a meeting environment following our model proposed. Thus, the experiment compared only two work environments: *SL with SLMeetingRoom* and *SL without SLMeetingRoom*.

The goal of the experiment was to assess the benefits to meetings when empowering the SL environment with meeting related components.

For the experiment, the groups were larger, the number of meetings increased and the task changed. The participants were the same as in the pilot study, thus ensuring that they were all warmed up, ready and fully engaged in all activities. As for the experiments internal validity, we should highlight that the groups’ readiness did not worsen their performance, as this was not the variable we were assessing.

#### Overview

The experiment consisted of a set of related work meetings, considering the two work environment settings: *SL with* and *SL without* the *SLMeetingRoom* components.

The experiment was carried according to the University’s guidelines, concerning software evaluations, does not require previous consent. Nonetheless, formal written consent were obtained from all participants before the experiment started. It was emphasized that, even though there was no harm in participating in the experiment, they could leave at anytime. Their participation was anonymous and we were looking for the aggregate behavior of the group. The experiment was in compliance with the Helsinki declaration.

#### Participants

The participants were the same as those of the pilot study, in a total of eleven people. They were allocated to the groups through a draw. Once again participants received no remuneration, but their scores acted as an incentive for participation.

#### Task

The task consisted of developing the final project of the HCI (Human-Computer Interaction) discipline. The deliverable: a simplified task model, user model, interface navigation model, interface storyboard and the final implemented and the heuristic evaluation questionnaire). Each group chose the project theme.

#### Duration

The groups were observed over a 30-day period. Each group held five meetings with a maximum duration of one hour. One group exceeded time stipulated by two minutes. Five meetings were held per group in a total of fifteen meetings.

#### Group and work space configuration

Three groups were formed through draws, one group with four participants and two groups with three participants. Two groups used the SLMeeting environment.

#### Process

As the participants had already taken part in the pilot study it was not necessary to ask them to respond to the user profile questionnaires. All meetings were video-recorded and annotated by an observer. The observer did not communicate with any participants of the groups. The meeting procedure was the same as the one adopted in the pilot study.

#### Data collected

The variables collected for each assessment criterion were the same as those used in the pilot study.

## Analysis and result

### Pilot study

#### Task completion

Data indicated that task completion was highly related to meeting agenda quality. The agenda not only structured the meeting, but also supported the preparation of meeting minutes that were distributed at the end of each meeting.

Objective measures revealed a high level of task completion in all groups. All groups managed to fulfill the proposed tasks. We believe that the high degree of task completion was achieved due to the advantages of using meeting agendas which, according to Niederman and Volkema (Niederman and Volkema, 1996), influence the quality of results, participants satisfaction and mainly time wasting.

We also verified that questionnaire answers related to participants perception regarding task completion was a faithful reflection of what in fact occurred during meetings considering task completion.

The *SL with SLMeetingRoom* group was the only one that exceeded the stipulated duration of one hour, thus compromising the fulfillment of one of the tasks on the meeting agenda. The participants of the SL without *SLMeetingRoom* group were very concerned not to exceed the stipulated duration of one hour, as they met in the meeting room in SL without any support component.

The groups that used audiovisual channels (Videoconference and *Face-to-Face*) had no problem administering meeting duration. These groups’ meeting duration were relatively low compared to the groups that used the SL environment. As (Gutwin and Greenberg, 1998), affirm, we believe that this occurs due to the speed and naturalness of obtaining information in an on-site context, where participants can see and hear the whole group.

#### Participation degree

A conversation turn begins when a participant starts to speak and lasts until he finishes (Kim et al., 2008). Thus, similarly to the scheme adopted by Sellen (Sellen, 1995), we defined a conversation turn as the time during in which a participant speaks, regardless of unsuccessful interruptions from other participants or overlapping speeches. A turn ends when a participant stops speaking, whether due to an interruption or a significant period of silence or a pause.

The participation degree metric was calculated as the ratio between total participant speaking time (DTCi) and total participant conversation turns (NTCi). Through this calculation and assuming a Gaussian distribution for its result, it was possible to identify that participants that are at the extremes of the Gaussian curve, i.e., persons who speak in only a few turns are many standard deviations above the mean, while those who speak in many turns will be many standard deviations below the mean. The Gaussian curve was used as a standard of comparison (baseline).

**Figure 3:**
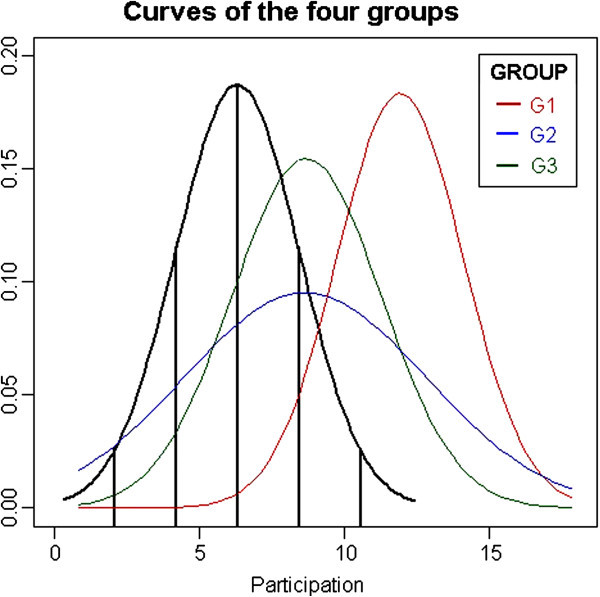
**Participation curves of each group compared with the Face-to-face group in the pilot study.**

An analysis of Figure 3 shows the differences in participations in meetings held by the different experimental groups. The Face-to-Face group had the lowest average level of participation, due to the fact that it was the group that used the most conversation turns (total of 1222 turns), thus resulting in a much lower average per turn. However, this was already expected, as audio/visual media are more dynamic and transmitted more rapidly and in many turns (Kirk et al., 2007).

The group that used the *SL with SLMeetingRoom* environment was the one whose meetings were the most distant from the curve of the Face-to-Face group, i.e. its level of participation was at the extremity of the *Face-to-Face* group’s curve (more than two deviations). It is important to observe that the curve of the *SL with SLMeetingRoom* group represented a shift of the *Face-a-Face* group’s curve. We believe that this occurred because the group chose the communication via text channel, i.e. the conversation turns were greater due to the time spent reading, formulating and typing content. The performances of the *Videoconference* and *SL without SLMeetingRoom* groups, on the other hand, were closer to the mean of the *Face-to-Face* group in all meetings.

The post-meeting questionnaires made it possible to assess participants’ actual feelings related to their participation during meetings. The *SL* withou*t SLMeetingRoom* was the one in which people felt really contributing to the task, followed by the *Face-to-Face*, *SL* withou*t SLMeetingRoom*, and finally by the *Videoconference* group. The *Videoconfer*enc*e* group was the one that felt the least committed to the goal, expressing exactly what was shown by the participation criterion, in which the group had the shortest conversation turn durations, or a total of 01 h 49 m 03 s during the four meetings.

#### Cognitive effort

**Figure 4:**
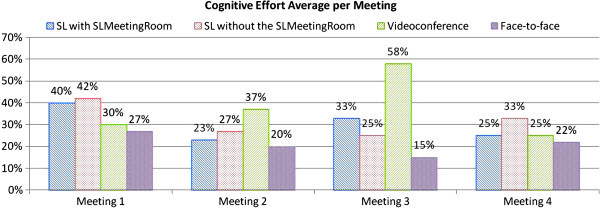
**Cognitive effort measured by the post-meeting questionnaire.**

Cognitive effort was calculated according to the weight of each answer given by participants in the post-meeting questionnaire. After analyzing the questionnaire data, we assess the cognitive effort of groups in each meeting (Figure 4).

We verified that the *Face-to-Face meeting style* group was the one that held meetings with the least cognitive effort. This is due to the ease and naturalness of on-site interactions (Qiu et al., 2009). In the case of this assessment criterion we expected that the audio and video environment provided by *Videoconferencing* would produce data that were close to the *Face-to-Face* group. However, we verified that the *Videoconference* groups data is statistically different from the *Face-to-Face* groups data, i.e. the participants of the *Videoconference* group reported that they had problems holding meetings. The transcriptions of the *Videoconference* group’s conversations showed that the participants had many problems with the high consumption of Internet bandwidth, as the connection is not one-to-one but one-to-many. We believe that these problems of connection and Internet bandwidth were the factors responsible for increasing the cognitive effort of holding meetings. When analyzing the meeting records’ transcripts, we verified that the percentage of cognitive effort per meeting was proportional to the connection problems faced by the group during that meeting. We conclude that communication bandwidth still impacts participants’ cognitive effort since they break their reasoning due to communication length.

#### Sense of presence

Sense of presence is based on identifying linguistic hints within participants’ dialogues (Kramer et al., 2006). Whenever the person speaks about the remote space in the same way he speaks about the local space, we can infer that it is a sign he feels within the remote space. For this research, linguistic analysis was applied to groups holding meetings using various means of communication and the results were compared to those recorded in (Kramer et al., 2006). In an attempt to find factors that could explain the variance in results, the questions in the post-meeting questionnaire related to sense of presence were submitted to a varimax rotation factor analysis. This solution revealed three factors that explained 76% of data variance. Factor 1, called *Objects* which indicates how participants refer to objects in the work space; Factor2, called *Environment*, which informs how participants refer to the work space, was able to explain 25% of data variability; and Factor 3, called *Group*, which informed how participants felt in relation to work colleagues, managed to explain 20% of data variability. Thus, the things participants say about the objects, the environment and other participants explain the difference between replies for each factor. Varimax factor analysis tests a hypothesis to verify whether the three factors are sufficient to understand data variation. A p-value of 0.018 was found and thus the model is significant (p ¡ 0,05). In order to investigate the feasibility of using linguistic variables as a predictor of presence, we performed a regression to predict presence based on linguistic variables. The regression for Factor 1 (objects) explained 16% of presence scores (R2 = 0,1677; F[7,40] = 1,151; p-value = 0,352). The regression for Factor 2 (environment) was a significant model, representing 27% of variance in presence scores (R2 = 0,2751; F[7,40] = 2,169; p-value = 0,058). Factor 3 (group) explained 9% of presence scores (R2 = 0,0976; F[7,40] = 0,618; p-value = 0,737). Finally we measured the correlation between linguistic variables and presence scores for each of the three factors. Presence scores were positively correlated with the use of the pronouns *you* and *I*. This is consistent with the theory that a greater presence makes remote participants feel as if they were together in the same environment (Kramer et al., 2006). Presence scores were also highly correlated with the use of local and remote deixis, suggesting that when participants feel present in a remote environment, they speak about this in the same way that they speak about the physical environment. The correlation performed between the linguistic variables and presence scores showed that the factors were complementary. Although the correlations found were not significant, we highlight that in (Kramer et al., 2006) it was used a sample of N = 148.

### Experiment

#### Task completion

Similarly to the pilot study it was possible to observe that all groups managed to perform the proposed task. Our results ratify that the use of a meeting agenda alters the quality of the results, the participants’ satisfaction and, mainly, the participants’ sense of group (Niederman and Volkema, 1996). The SL without *SLMeetingRoom* group exceeded the meeting time stipulated, compromising the fulfillment of tasks in the two meetings. The analysis of the post-meeting questionnaires showed that, similarly to the pilot study, task completion results did not vary.

#### Level of participation

**Figure 5:**
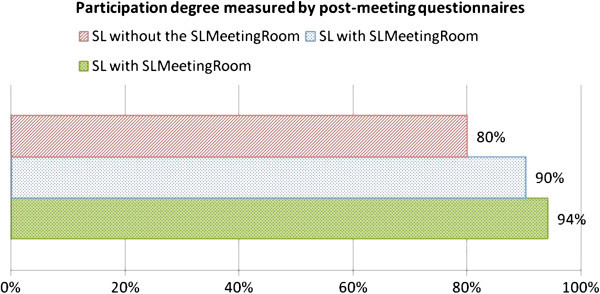
**Participation measured by the post-meeting questionnaire in the experiment.**

The degree of participation was calculated in the same way as in the pilot study, i.e. by the ratio between the speaking time of each participant and the number of conversation turns. The theoretical Gaussian curves based on the mean and standard deviation of each meeting were constructed and used as tools in order to understand the differences between meetings and groups. We measured the differences in the degree of participations according to each meeting setting. In general, the average degree of participation and the standard deviation increase during meetings, thus widening the curves. At the beginning participants speak longer and take few turns, but as the meeting goes on they may speak briefly but with a great number of turns. Group 3 (SL with *SLMeetingRoom* environment. setting) presented the highest degree of participation. Group 3 was followed by group 2 (SL without *SLMeetingRoom* environment setting) and, finally, by group 1 (SL with *SLMeetingRoom* environment setting). The post-meeting questionnaire (Figure 5) measured participants’ perception regarding their individual participation during meetings. We verified that the two groups in the SL with *SLMeetingRoom* environment felt more participative than the SL without *SLMeetingRoom*.

#### Cognitive effort

**Figure 6:**
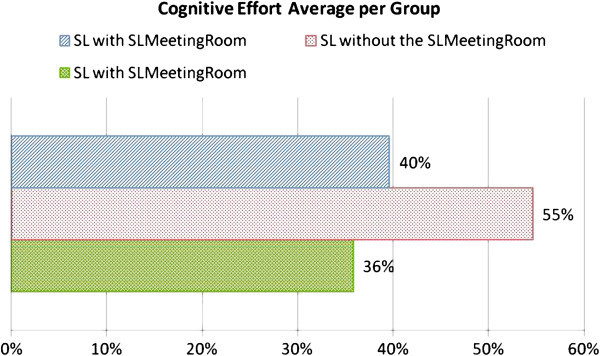
**Cognitive effort per meeting measured by the post-meeting questionnaire.**

An analysis of the post-meeting questionnaire data revealed groups’ cognitive effort during meetings, as shown in Figure 6. We verified, through the questionnaires, that the groups within the *SLMeetingRoom* environment made a lower cognitive effort. This reinforces our research hypothesis that the groups that used Second Life allied to the *SLMeetingRoom* components model would make a lower cognitive effort during meetings. According to transcriptions of conversations, the participants that used the *SLMeetingRoom* model in the pilot study and the environment without any components in the experiment, reported problems using SL without *SLMeetingRoom* to support meetings.

##### Revised transcription: meeting 1 group 2 -SL without SLMeetingRoom 05/31/2010

*P1: Why dont we have the environment?*

*P2: Why dont we have any meeting object? :(((*

*P2: The room with objects is more interesting.....*

*P2: Wheres the chronometer? :(((( And the meeting agenda? Where are we going to put it?*

*P1: Its in Google docs.*

*P2: I know, but we always bring it to the meeting and put it on the white board.*

*P2: Its really bad without the environment. We have to keep looking at the watch and count time. And theres nowhere to put the minutes and the meeting agenda on the white board. I miss all this.*

*P2: Holding a meeting with no white board with the agenda is more complicates things and also not having a schedule to list who is going to do what. I liked the meeting environment very much.*

##### Revised transcription: meeting 2 group 2 -SL without SLMeetingRoom 06/06/2010

*P2: Another thing. When someone speaks, there is no way of knowing whether he is still writing. The text of one person overlaps with that of another who speaks more..*

*P3: I think this due more to the delay.*

*P2: I don’t think so.*

*P3: Sometimes, mine took a long time to post here.*

*P2: I think things worked better when I used Social Proxy.*

*P3: Social Proxy certainly helps.*

*P3: Social Proxy helps one to see that the other person is still writing.*

*P1: I only used it without the environment. The results were bad.*

*P2: I used it with the environment. It was really better.*

An analysis of the conversation transcripts shows that group2 participants missed the meeting support components of *SLMeetingRoom* model. The need for the work group components in the environment was detected in answers of the post-meeting questionnaire answers related to high cognitive effort (75%). We conclude that the cognitive effort is greater when participants do not meet in an environment prepared for meetings.

#### Sense of presence

As in the pilot study, questions regarding sense of presence were also submitted to a varimax rotation factor analysis. A single factor explains 45% of data variability. After using a varimax rotation factor analysis we tested the hypothesis that only one factor is sufficient to explain data variability. The research found a p-value of 2,63 and 0,06, therefore the model is significant (p ≤ 0, 05). As in the case of the pilot study, in order to investigate the feasibility of using linguistic variables as a predictor of presence, we performed a regression to predict presence based on linguistic variables. The regression model for the factor was significant (pvalue ≤ 0,05), explaining 30% of presence scores variability (R2 = 0,307; F[7,47] = 2,986; p-value = 0,011). Our results were similar to the results obtained by Kramer (Kramer et al., 2006), who has a sample of N = 148 and was able to represent 33% of the variance of presence scores. Finally we measured the correlation between linguistic variables and presence scores for the factor found. Presence scores were positively correlated with the use of the pronouns we and negatively correlated with the use of pronouns like you and I. This is consistent with the theory that a greater presence makes remote participants feel as if they were together in the same environment (Kramer et al., 2006). Presence scores were also highly correlated with the use of local and remote deixis, suggesting that when participants feel present in a remote environment, they speak about this in the same way as they speak about their physical environment. None of the experiments showed significant correlations and we believe that this may be due to misuse of language, for example during moments when a participant used the pronoun you when he should in fact have used we.

### Hypothesis test

The hypothesis test was used in order to find statistical evidence of differences between groups. After applying the Jonckheere-Terpstra test (Hollander and Wolfe, 1973), we obtained a p-value for each assessment criterion. We then verified whether the significance of the result obtained per p-value was below or above 5%. We consider that if the p-value is below 0,05 there is no evidence in favor of *H*1, i.e., we should accept *H*0, and reject otherwise.

The results for the task completion criterion did not vary, so no further test was done.

#### Pilot study

Each group, participating in the experiment, held the project meetings using one of the work environments: *Face-to-Face*, *Videoconference*, *SL with SLMeetingRoom* and *SL without SLMeetingRoom*. In order to facilitate comparison we refer to the groups as G1, G2, G3 and G4, respectively. The results of the pilot study are shown in Table 2.

**Table 2:** **Hypothesis test with the pilot study data**

Assessment Criteria	***H***0	***H***1	JT: p-value	Decision
Task Completion	. . .	. . .	. . .	. . .
Degree of Participation	*G*1 = *G*2 = *G*3 = *G*4	*G*2 ≤ *G*3 ≤ *G*1 ≤ *G*4 ou *G*2 ≤ *G*1 ≤ *G*3 ≤ *G*4	0,87	Accept *Ho*
Cognitive Effort	*G*1 = *G*2 = *G*3 = *G*4	*G*2 ≥ *G*3 ≥ *G*1 ≥ *G*4 ou *G*3 ≥ *G*2 ≥ *G*1 ≥ *G*4	0,007	Reject *H*0
Sense of Presence	*G*1 = *G*2 = *G*3 = *G*4	*G*2 ≤ *G*3 ≤ *G*1 ≤ *G*4 ou *G*3 ≤ *G*2 ≤ *G*1 ≤ *G*4	*Factor*1 = 1, 68*E* − 6	Reject *Ho*

As shown in Table 2, cognitive effort and sense of presence results indicated our hypothesis holds, i.e. *SLMeetingRoom* presents the closest results to face-to-face meeting settings.

However, the degree of participation does not seem to be affected by the meeting setting. As shown by the data, no matter the setting, the degree of participation of the group was similar. Hence, the null hypothesis cannot be rejected. There are some feasible explanations for this behavior. Since the students knew they would be graded and that the experiments were been recorded, they might have been afraid that degree of participation would have influenced their final grade. Consequently, no matter the meeting setting, they wanted to register an active role in the meetings. Another possible reason would be the combination of the small size of the groups and the short duration of the experiment for grading. This combination might have put pressure in all participants to work harder.

Our pilot study confirmed that face-to-face meeting is the best meeting setting for group work, whenever possible considering time and participants’ location feasibility. Our study also suggested that video conferencing setting works properly, but with drawbacks as far as sense of presence and degree of participation are concerned. Second life environment shares the same features and drawbacks outlined for videoconferencing technology. The question remains if it is possible to surpass these drawbacks, enriching Second Life environment with a set of pre-defined elements tuned to support group work without imposing too much extra efforts burden on users. Our posterior experiment focused only on comparing meetings done in these two later settings.

#### Experiment

Each group held project meetings in one of the two work environments (with or without *SLMeetingRoom*). In order to facilitate comparison we refer to the groups as G1 and G3 (SL with *SLMeetingRoom*), and G2 (SL without *SLMeetingRoom*).

The results, shown in Table 3, were similar to the ones found during the pilot study confirming that *SLMeetingRoom* set up presented a better sense of presence and lower cognitive effort than using standard Second Life environment. Degree of participation was also not affected by the meeting settings. We believe the same possible reasons for the pilot study results apply.

**Table 3:** **Hypothesis test with the experiment’s data**

Assessment Criteria	***H***0	***H***1	JT: p-value	Decision
Task Completion	. . .	. . .	. . .	. . .
Degree of Participation	*G*1 = *G*2 = *G*3	*G*2 ≤ *G*3 ≤ *G*1 ou *G*2 ≤ *G*1 ≤ *G*3	0,794	Accept *Ho*
Cognitive Effort	*G*1 = *G*2 = *G*3	*G*2 ≥ *G*3 ≥ *G*1 ou *G*2 ≥ *G*1 ≥ *G*3	0,017	Reject *H*0
Sense of Presence	*G*1 = *G*2 = *G*3	*G*2 ≤ *G*3 ≤ *G*1 ou *G*2 ≤ *G*1 ≤ *G*3	*Factor*1 = 0,015	Reject *Ho*

It is interesting to observe that Task completion was also not impacted by the meeting set up. This might have happened because the task was doable in the stipulated time frame and because task completion was for the final grade.

## Conclusion

This paper presented a study using Second Life as an environment for holding meetings. Given that Second Life was not created with EMS characteristics, we designed an enriched environment, called the *SLMeetingRoom* model, which is composed of ten essential meeting support functionalities to cover communication, coordination, cooperation and perception needs of working in groups.

We postulated the hypothesis that using *SLMeetingRoom* to support work meetings produces results that are closer to those achieved in face-to-face interactions than other methods.

We performed a comparative experimental study, with computer science graduate students, in two phases: pilot study and experiment. The pilot study compared groups working on four different group-meeting settings, including face-to-face, video conferencing, traditional Second Life environment and Second Life with *SLMeetingRoom* components. The experiment focused only in the two last settings. Although all students had no prior experience with Second Life environment, they were computer science students, so cognitive effort results might be biased.

We collected evidences that the *SLMeetingRoom* is a promising group-meeting environment. It maintains required low cognitive effort from users, allowing them to deal with the technology while presenting a higher sense of presence of the team members than the standard SL meeting environment. In our experiments, degree of participation and task completion were not affected by the meeting set up.

This research emphasized the advantages of using the Second Life environment, augmented with meeting support components, as an EMS tool, helping groups to reach agreements and to establish targets, thus making collaborative meetings more efficient.

In addition to the limitations concerning the group composition (CS students) and the type of task (design), our studies were limited to meetings of small groups, to develop tasks in a specified number of meetings (four) and with a defined reward (grade).

In addition to the experiments, this study raised a series of interesting questions regarding the criteria for evaluating meetings that should go beyond task completion and degree of participation, but should also include the sense of presence that makes a group, a team and the cognitive effort required to use any meeting support technology.

To overcome the limitations of our case studies, we are planning new experiments involving larger groups and with a more varied participants’ profiles (level of academic training, occupation, domain area.).

Second Life possesses characteristics peculiar to 3D virtual environments that offer advantages in relation to other platforms, such as a high level of immersion, great sense of presence in the group and environment, ability to contextualize a meeting environment with the participants sitting around a table, ease of synchronous communication (textual, oral, gestural), high perception of the activities of group participants, amongst others. We conclude, therefore, that Second Life is a good tool for holding remote synchronous meetings and can be used alongside videoconferencing, EMS, audio-conferencing and screen sharing.

### Ethical approval

The study was carried according to the University’s guidelines, concerning software evaluations, does not require previous consent. Nonetheless, formal written consent were obtained from all participants before the experiment started. It was emphasized that, even though there was no harm in participating in the experiment, they could leave at anytime. Their participation was anonymous and we were looking for the aggregate behavior of the group. The experiment was in compliance with the Helsinki declaration.

### Consent

Written informed consent was obtained from all participants for publication of this report and any accompanying images of their avatars.

## Endnote

^a^Property of some linguistic elements such as personal and demonstrative pronouns (this, there, here, there, that, etc.)(de Holanda Ferreira, 1999).

## Acknowledgement

The authors would like to thank CAPES-Brazil and CNPq-Brazil for their financial support. We also should be grateful to all students of the Interface and Multimedia’ 2010 and 2011 for their participation in the experiments.
